# Lactate causes downregulation of *Helicobacter pylori* adhesin genes *sabA* and *labA* while dampening the production of proinflammatory cytokines

**DOI:** 10.1038/s41598-022-24311-5

**Published:** 2022-11-21

**Authors:** Tanvi Somiah, Hanna G. Gebremariam, Fanglei Zuo, Ksenija Smirnova, Ann-Beth Jonsson

**Affiliations:** Department of Molecular Biosciences, The Wenner-Gren Institute, Stockholm University, Svante Arrheniusväg 20C, 10691 Stockholm, Sweden

**Keywords:** Microbiology, Pathogens

## Abstract

Chronic inflammation induced by *Helicobacter pylori* is strongly associated with gastric cancer development, which is influenced by both bacterial virulence and host genetics. The sialic acid-binding adhesin SabA and the MUC5AC-binding adhesin LabA are important *H. pylori* virulence factors that facilitate adhesion of the bacterium, which is a crucial step in colonization. Lactate utilization has been reported to play a key role in the pathogenicity of different bacterial species. However, this is poorly understood in *H. pylori*. In this study, we investigated the effect of lactate on *H. pylori* adhesin gene expression and the regulation of host inflammatory cytokines. We show that the bacterial adhesins SabA and LabA were downregulated at the transcriptional level during incubation of *H. pylori* with lactate. Downregulation of *sabA* required the involvement of the two-component system ArsRS, while *labA* was regulated via the CheA/CheY system, indicating differences in the regulation of these genes in response to lactate. The levels of the proinflammatory cytokines TNF and IL-6 in *H. pylori*-stimulated macrophages were reduced when lactate was present. Interestingly, glucose did not prevent the secretion of these cytokines. Taken together, our data suggest that lactate affects *H. pylori* adhesin gene expression and the host response upon infection.

## Introduction

*Helicobacter pylori* is a human-specific gastric pathogen capable of successfully colonizing in a biological niche in which few other microorganisms are able to colonize: the harsh gastric environment of the human stomach. *H. pylori* infection can remain asymptomatic for several years; however, long-term infection has been linked to the development of gastroduodenal ulcers and gastric cancer in certain individuals^1^. To overcome the highly unhospitable factors in this region, such as the highly acidic pH of the gastric lumen and the constant turnover of the mucous layer, *H. pylori* must deploy a range of well-timed virulence factors in the absence of which this pathogen would simply be killed or washed away by the host^2^. One important subset of these factors is several outer membrane bacterial proteins OMPs known as adhesins. While these adhesins have been poorly categorized in the past, advances in science and the development of transgenic animal models that carry defined human pathogen receptors have been instrumental in identifying several of these proteins and understanding their role in pathogenesis^3,4^.

BabA binds to fucosylated Lewis^b^ (Le^b^) antigens found on the surface of gastric epithelial cells, and its evolution has helped *H. pylori* to adjust to the individual host mucosal glycosylation that it encounters upon infection^4^. Another well-studied adhesin is sialic-acid binding adhesin (SabA), which can bind to the sialyl-Lewis^x^ (Le^x^) antigen found on gastric epithelial cells and neutrophils. SabA is regulated by phase variation in response to environmental cues, such that *sabA* can be rapidly switched ‘on’ or ‘off’ to adapt the bacteria to changes exerted by the gastric niche^5^. BabA may also be subjected to rapid on/off shift depending on which loci the *babA* gene is located in^6–8^. Both SabA and BabA can be switched on or off via gene conversion^6,7,9^. The sialylated Le^x^ antigen that serves as a receptor for SabA is poorly expressed in healthy mucosa but is upregulated under inflammatory conditions^10^. Accordingly, the importance of SabA in adhesion could come to the forefront in an ongoing *Helicobacter pylori* infection in which the mucosa is inflamed^11^, in contrast to early expressed BabA which assists in initial colonization. One of the more recently discovered mucin-binding adhesins, LabA, is known to bind the N,N′-diacetyllactosediamine (LacdiNAc) motif that is expressed on the surface of MUC5AC gastric mucins^12^, whose expression is restricted to the gastric mucosa, which could explain the tissue tropism of *H. pylori.* Among 72 seropositive *H. pylori* patients tested, more than 99% of the bacteria were found to be associated with the MUC5AC mucin, indicating that *H. pylori* is very closely associated with this mucin^13^.

The ability of bacteria to thrive in the biological niches of their host depends heavily on their ability to sense and respond to external stimuli. Primarily, this job is fulfilled by the specialized signal transduction mechanisms which rely on two component systems. Each two component system comprises of a sensor histadine kinase (HK) component and the response regulator component (RR). One way of defining the cascade of signalling events that occur when a TCS receives an external signal is by dividing the sequence of events into four main areas, (1) extracellular signalling^14^, autophosphorylation of the HK component, (3) phosphotransfer and lastly (4) regulator response^15^ and can be explained as follows: external stimuli such as pH change, nutrients, osmotic pressure, quorum sensing proteins and antibiotics^16^ activates the HK component and causes its autophosphorylation. The phosphporylated HK in turn transfers a phosphoryl group to the RR, and lastly the phosphorylation of the RR component causes changes in its biochemical properties, thereby leading it to carry its final output functions such as DNA binding and transcriptional regulation, protein–protein interaction or enzymatic activities^17^.

The two component systems (TCS) repertoire of *H. pylori* can be said to be much smaller than that of several of its bacterial counterparts, having only four complete sensor histidine kinase–response regulator pairs (SK-RRs)^18,19^. Consequently, some TCSs control a large number of genes in response to a variety of external stimuli. For example, the most well-studied TCS in *H. pylori* is ArsRS (acid-response signaling), having acidic pH as the key environmental signal. The ArsRS TCS comprises of a histidine kinase HP0165 and response regulator HP0166 and earlier studies revealed that HP0166 negatively autoregulates its own expression along with acting as a transcriptional activator of several *H. pylori*-specific genes whose functions were unknown at the time^20^. Currently the TCS ArsRS is known to control over 100 genes^21^, including *sabA* repression via the ArsR-DNA interaction in response to low pH^22^. Regulation via this system involves autophosphorylation of the membrane-bound histidine kinase component ArsS, which in turn causes phosphorylation of the response regulator ArsR that binds to the promotor regions of target genes and thereby causes their transcriptional repression^21^. CheA and CheY make up a two-component regulatory system in which CheA regulates phosphorylation of the final output protein CheY, which in turn modulates flagellar rotation via protein‒protein interaction with the flagellar motor switch complex^23,24^. *cheY* mutants of *H. pylori* strain N6 have been shown to be nonmotile in vitro and show an inability to colonize in mice, although they are able to generate an initial immune response from the host^24^. There is mounting evidence of the multifaceted roles that *H. pylori* TCSs play beyond regulation of genes essential for bacterial viability, such as the possibility of adaptation of metabolic processes within the bacteria in response to environmental stimuli^25^.

Lactate in the body can be found in its two isomeric forms, known as l- and d-lactate. While the human body predominantly produces l-lactate, lower microorganisms, such as bacteria in the gut, are known to produce and utilize both forms^26^. The effect of lactate in relation to virulence has been studied in several different microorganisms; for example, *C. albicans* are capable of masking β-glucan, a well-known pathogen-associated molecular pattern (PAMP), in response to lactate, thus reducing its visibility to the host^27^. *Neisseria meningitidis* is known to utilize lactate to withstand complement-mediated lysis, which has been shown through the loss of resistance against complement-mediated killing in lactate permease (lctP) mutants^28^.

On the other hand, thus far, little has been found concerning the relationship between *H. pylori* virulence and lactate. Previous studies have shown that l-lactate produced by gastric epithelial cells can enhance the growth of *H. pylori,* and in 2017, l-lactate was shown to be a chemoattractant for *H. pylori* sensed via the lactate chemosensor TlpC^29,30^. In this study, we aimed to determine whether l-lactate can affect the expression of certain adhesins used by *Helicobacter pylori* to successfully bind to gastric epithelial cells of the host, as well as the underlying molecular mechanisms behind their regulation.

## Results

### Lactate inhibits expression of the *H. pylori* adhesin genes *sabA* and *labA*

**Figure 1:**
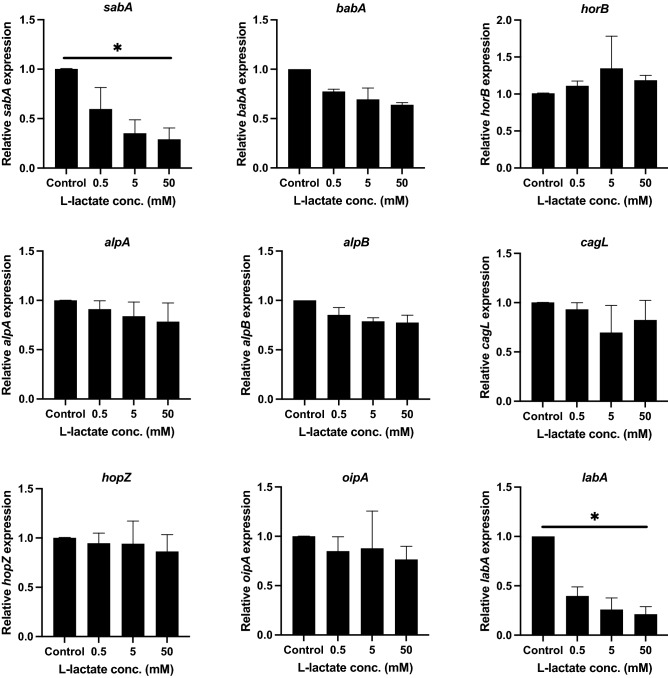
l-lactate inhibits *sabA* and *labA* expression in *H. pylori.* Gene expression of *H. pylori* adhesins after incubation of the bacteria in RPMI 1640 medium without glucose (control) or supplemented with various concentrations of l-lactate. The mRNA expression levels of various *H. pylori* adhesin genes were quantified using qPCR after 2 h. Target mRNA levels were normalized to those of the housekeeping gene *gyrB*. Gene expression levels in the control were set to 1. Data represent the means and standard deviation of duplicate samples and are average of three independent experiments. *P < 0.05; ns, nonsignificant, using ANOVA followed by a Bonferroni posttest.

To investigate the effect of lactate on adhesion factors of *H. pylori* 67:21*,* we performed an initial screening of several important adhesin genes in the presence of lactate via qPCR. The l-lactate produced by gastric cells has been reported to reach a concentration of 0.3–1 mM in gastric juice^30^, and although the stomach distribution of lactate is not known, the concentration could be even higher in the region closer to the cells where it is produced. Additionally, the microflora in this region contribute to lactate production in the gut. Hence, in this experiment, we chose to test lactate concentrations of 0.5 mM, 5 mM and 50 mM. The expression of most of the adhesins was not significantly affected by l-lactate; however, there was a significant downregulation of *sabA* and *labA* at all concentrations of lactate tested (Fig. 1). This effect was true not only for l-lactate, which is the form of lactate that is predominantly produced by host cells, but was also seen for d-lactate (Fig. S1), which is mainly produced in the body by fermentative microorganisms, such as lactobacilli. To further confirm the effect of lactate, we tested two additional strains, 26695 and J99, which both showed similar reduced *sabA* and *labA* epxpression as strain 67:21 (data not shown).

**Figure 2:**
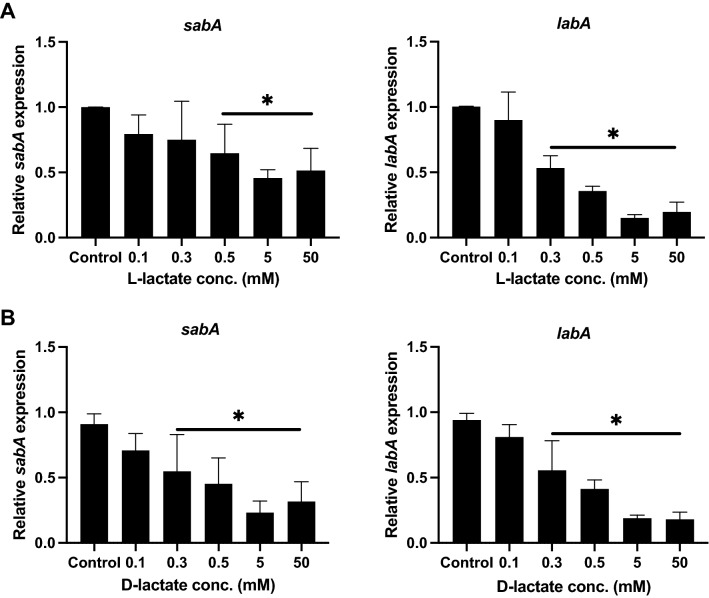
Gene expression of *sabA* and *labA* in the presence of different concentrations of l- and d-lactate. Relative expression levels of the *H. pylori* adhesin genes *sabA* and *labA* after incubation of the bacteria in RPMI 1640 medium without glucose (control) or supplemented with various concentrations of either l-lactate (**A**) or d-lactate (**B**) for 2 h. Target gene expression was normalized against that of the housekeeping gene *gyrB*. Gene expression levels in the control were set to 1. Data represent the means and standard deviation of duplicate samples and the average of three independent experiments. *P < 0.05; ns, nonsignificant, using ANOVA followed by a Bonferroni posttest.

Further analysis of *sabA* and *labA* expression in both 0.1 mM and 0.3 mM lactate revealed that physiologically relavant levels of 0.3 and 0.5 mM l-lactate are required to significantly downregulate *labA* and *sabA* respectively, while 0.3 mM d-lactate and above is enough to cause a reduction in expression of both *sabA* and *labA*, below which no significant change was observed (Fig. 2). Based on these results, we sought to investigate whether this effect was unique to lactate or whether it was a general response to other sugars. For this reason, we tested fructose, an intermediate sugar in the glycolysis pathway that some strains of *H. pylori* utilize even preferentially over glucose as a carbohydrate source^31^. Additionally, we also tested butyrate, an *H. pylori* nonmetabolite mainly produced by certain gut microbiota via the fermentation of dietary fibers^32^. Interestingly, we did not observe the same effect on *sabA* and *labA* with these two substances, indicating that the downregulation is a specific response to lactate (Fig. S2).

### The lactate chemosensor TlpC is not involved in *sabA* and *labA* downregulation

**Figure 3:**
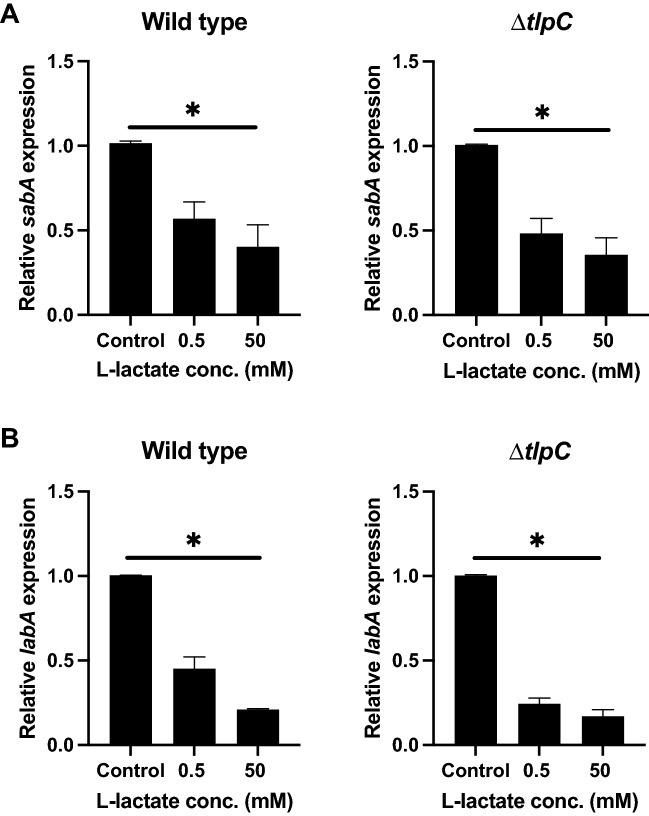
The lactate chemosensor TlpC is not involved in *sabA* and *labA* downregulation. Relative expression levels of the *H. pylori* adhesin genes *sabA* (**A**) and *labA* (**B**) in wild-type and ∆*tlpC* mutant *H. pylori* after incubation of the bacteria in RPMI 1640 medium without glucose (control) or supplemented with 0.5 mM or 50 mM of l-lactate for 2 h. Target gene expression was normalized against that of the housekeeping gene *gyrB*. Gene expression levels in the control were set to 1. Data represent the means and standard deviation of duplicate samples and the average of three independent experiments. *P < 0.05; ns, nonsignificant, using ANOVA followed by a Bonferroni posttest.

Based on the results showing that *sabA* and *labA* downregulation was lactate specific, we hypothesized that the regulation could occur through the sensing of lactate by the *H. pylori* chemosensor TlpC. We incubated both the wild type and the Δ*tlpC* mutant in 0.5 mM and 50 mM of lactate for 2 h and then performed qPCR to determine the mRNA expression levels of the genes of interest. We found that the wild type behaved similarly to the Δ*tlpC* mutant in the presence of lactate, showing the same differences in gene expression. Both the wild type and the Δ*tlpC* mutant showed significant downregulation of *sabA* and *labA* in the presence of 0.5 and 50 mM lactate (Fig. 3A,B).

### Lactate-mediated downregulation of *sabA* expression is dependent on the ArsRS two-component system

**Figure 4:**
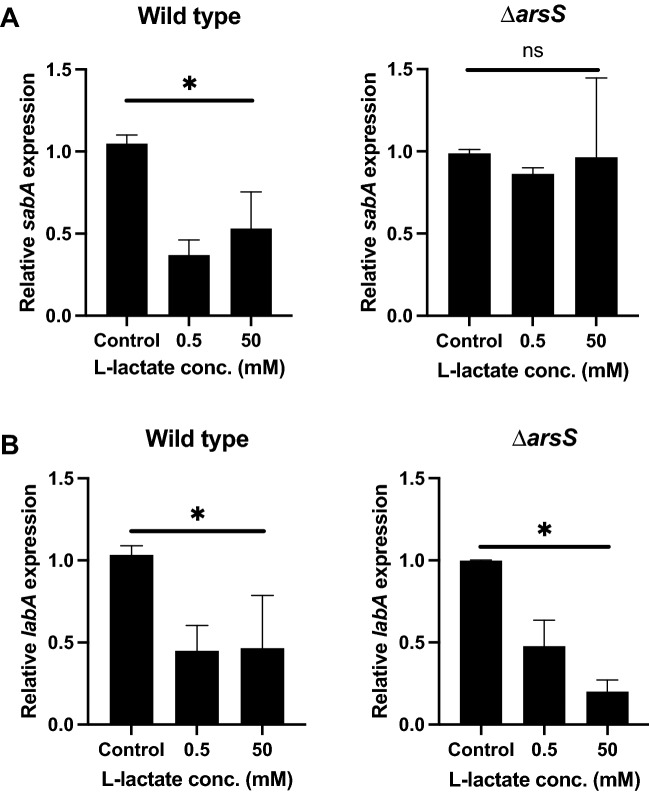
The lactate-mediated downregulation of *sabA* expression is dependent on the ArsRS two-component system. Relative expression levels of *sabA* (**A**) and *labA* (**B**) in wild-type and ∆*arsS* mutant *H. pylori* after incubation of the bacteria in RPMI 1640 medium without glucose (control) or supplemented with 0.5 mM or 50 mM l-lactate for 2 h. Target gene expression was normalized to that of the housekeeping gene *gyrB*. Wild-type and ∆*arsS* mutant bacteria were analyzed with their respective controls set to 1 to determine the relative values of *sabA* and *labA* expression in each of the samples incubated with lactate. Data represent the means and standard deviation of duplicate samples and are an average of three independent experiments. *P < 0.05; ns, nonsignificant, using ANOVA followed by a Bonferroni posttest.

Since we did not observe a difference in the effect of lactate between the wild type and the Δ*tlpC* mutant, we were interested in checking whether any of the functionally diverse *H. pylori* two-component systems were involved in the regulation of the genes of interest. We first assessed the involvement of the ArsRS two-component system by incubating both the wild type and an ∆*arsS* mutant in the presence of 0.5 and 50 mM of lactate, followed by qPCR to check gene expression. Interestingly, we found that *sabA* was no longer downregulated in the absence of a functional ArsRS system (Fig. 4A), while *labA* still showed the same downregulation in the wild type and mutant (Fig. 4B). The ArsRS system has been known to respond to changes in pH; thus, we checked whether any of the added concentrations of lactate changed the pH of the medium to eliminate the possibility that the observed downregulation was an outcome of this change. Compared to the control, no pH change was observed for any of the lactate concentrations tested (Fig. S3).

### LabA is regulated differently than *sabA* in the presence of lactate via the CheA/CheY two-component system

**Figure 5:**
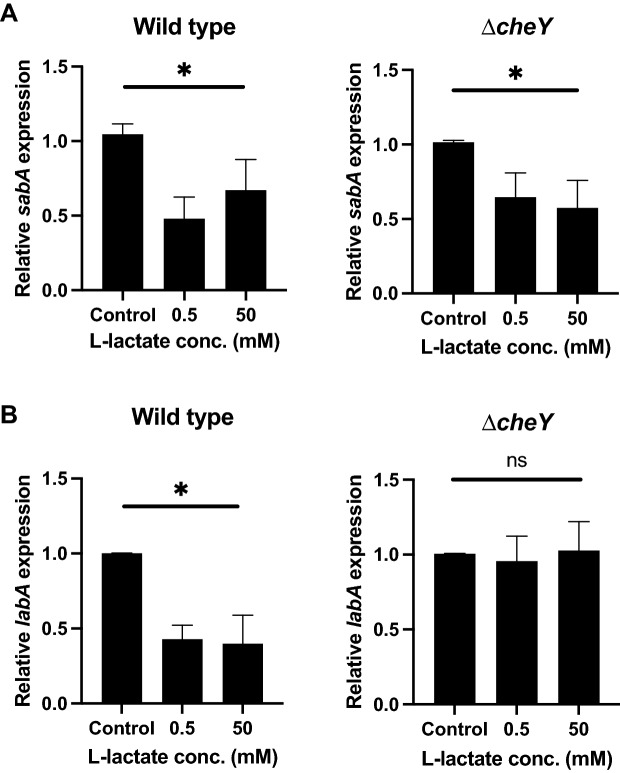
LabA is regulated differently than *sabA* in the presence of lactate via the CheA/CheY two-component system. Relative expression levels of *sabA* (**A**) and *labA* (**B**) in wild-type and ∆*cheY* mutant *H. pylori* after incubation of the bacteria in RPMI 1640 cell culture medium without glucose (control) or supplemented with 0.5 mM or 50 mM l-lactate for 2 h. Target gene expression was normalized to that of the housekeeping gene *gyrB*. Wild-type and ∆*cheY* mutant bacteria were analyzed with their respective controls set to 1 to determine the relative values of *sabA* and *labA* expression in each of the samples incubated with lactate. Data represent the means and standard deviation of duplicate samples and are an average of three independent experiments. *P < 0.05; ns, nonsignificant, using ANOVA followed by a Bonferroni posttest.

To further elucidate the mechanism behind *labA* downregulation, we incubated wild-type *H. pylori* and the Δ*cheY* mutant in the presence of 0.5 and 50 mM of lactate for 2 h. Here, we found that the regulation of *labA* involved the CheA/CheY two-component system, since the downregulation of *labA* was lost when *cheY* was deleted from the bacteria (Fig. 5B). On the other hand, *sabA* did not appear to be under the control of CheA/CheY, since both the wild type and Δ*cheY* showed the same differences in *sabA* expression in the presence and absence of lactate (Fig. 5A).

### Lactate dampens the production of the proinflammatory cytokines TNF and IL-6 in *H. pylori*-infected macrophages, while glucose has the opposite stimulatory effect on their production

**Figure 6:**
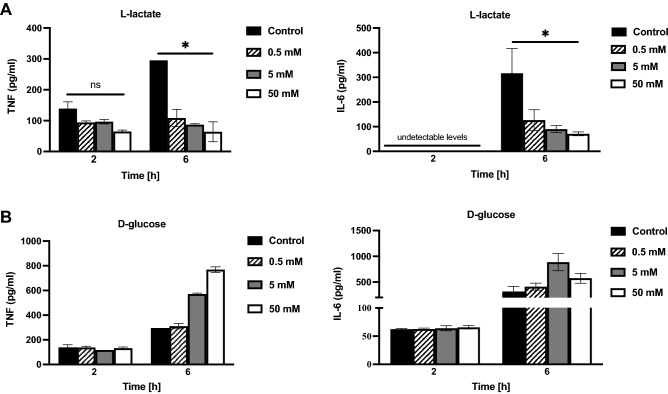
l-Lactate but not d-glucose reduces TNF and IL-6 production in macrophages. THP-1 cells were stimulated with *H. pylori* alone (control) or in combination with different concentrations of l-lactate (**A**) or d-glucose (**B**) for 2 and 6 h. The amounts of TNF and IL-6 in the cell-free supernatants were determined by ELISAs. Data represent the means and standard deviation of triplicate samples and are representative of two independent experiments. *P < 0.05 using ANOVA followed by a Bonferroni posttest.

For decades, lactate production in host cells has been considered to be nothing more than a metabolic byproduct. However, recent studies have shown that lactate plays a much larger role as a complex immunomodulatory molecule that controls how immune cells respond to infections in the tissue microenvironment^33^. *Mycobacterium tuberculosis*-infected macrophages have been shown to switch from pyruvate oxidation to a reduction of pyruvate into lactate, further causing an increase in the production of anti-inflammatory IL-10 as a result of this metabolic switch^34^. Additionally, changes in *H. pylori* adhesin expression have been shown to be linked to a change in cytokine production by host cells; the binding of the blood group antigen binding adhesin BabA to Lewis b (Le^b^) blood group antigens of epithelial cells triggers increased production of the pro-inflammatory cytokine IL-8 in Le^b-^positive cells compared to infection with *babA* deletion mutants^35^. Since we found a downregulation of *H. pylori* adhesins in the presence of lactate, we were curious to investigate the immune responses of host macrophages to the same molecule and to examine whether these changes could work in favor of the bacteria or against it. We therefore investigated the production of the proinflammatory cytokines TNF and IL-6 in *H. pylori-*infected macrophages in the presence and absence of lactate by incubating them together for 2 h and 6 h and then performing ELISAs. *H. pylori* in the absence of added lactate increased the secretion of both TNF and IL-6 in a time-dependent manner (Fig. 6A, black bars). However, when the cells were infected with *H. pylori* together with different concentrations of lactate, the production of both TNF and IL-6 was reduced compared to that in the control without lactate (Fig. 6A). Since *H. pylori* utilizes l-lactate as a carbon source, we tested d-glucose as an alternative carbon source to examine whether the reductions in cytokine production were merely due to metabolic effects of the bacteria. However, glucose was unable to bring about the same dampening of cytokine production and instead had an opposite stimulatory effect that led to increased levels of cytokine production at 6 h postinfection (Fig. 6B). The possibility of the host immune response being a result of metabolic differences in the bacteria in different media can further be ruled out by the growth curve results, which show that *H. pylori* grows similarly in medium containing l-lactate, d-glucose or neither supplement (Fig. S4).

### The phagocytic ability of *H. pylori*-infected macrophages remains unaltered by the presence of lactate

**Figure 7:**
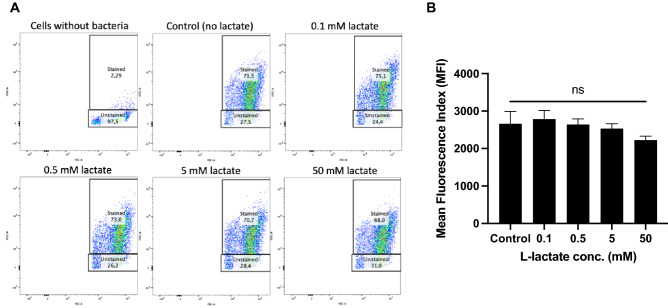
The phagocytic ability of *H. pylori*-infected macrophages remains unaltered by the presence of lactate. THP-1 cells were stimulated with CFSE-stained *H. pylori* alone (control) or in combination with different concentrations of l-lactate for 6 h. The number of phagocytosed bacteria was measured by flow cytometry (**A**). The mean fluorescence index (MFI) of the cells was also measured, confirming that there was no change in the phagocytosis of *H. pylori* in the presence of lactate (**B**). Data represent the means and standard deviation of triplicate samples and are representative of three independent experiments. ns, nonsignificant, using ANOVA followed by a Bonferroni posttest.

Lactate signaling has been shown to directly shape the activation, proliferation and metabolic activity of immune cells^33^; for example, macrophages in the presence of lactate can be induced to polarize to M2 macrophages by sensing lactate via the gpr132 receptor^36^, and M2-like macrophages have been shown to exhibit a higher phagocytic ability toward *E. coli* and cancer cell targets than M1-like macrophages^37^. Here, we were interested in studying whether lactate can cause a change in the phagocytic ability of *H. pylori*-infected macrophages, in addition to the changes in cytokine production that we observed earlier. We therefore infected macrophages with *H. pylori* for 6 h in the absence or presence of different concentrations of lactate and then measured the amount of CFSE-stained, internalized bacteria using flow cytometry. Interestingly, the phagocytic ability of the macrophages was not impaired by the presence of lactate, since the bacteria were phagocytosed by approximately 70% of the cells in all the samples tested (Fig. 7A). Additionally, we compared the mean fluorescence index (MFI) of the samples because single cells are capable of phagocytosing more than one bacterium. Again, we noted no differences between the MFI of the control and that of the samples containing lactate (Fig. 7B), indicating that changes in the cytokine production of the macrophages were independent of their ability to engulf *H. pylori*.

## Discussion

Lactate has been shown to play a key role in the pathogenicity of various microorganisms^38^. In this study, we examined the role of lactate in *H. pylori* pathogenesis and showed that both l- and d-lactate inhibit the expression of *H. pylori* adhesin genes and the production of *H. pylori*-induced proinflammatory cytokines. Furthermore, the two-component ArsRS system was required to accomplish the downregulation of *sabA* in the presence of lactate, while the downregulation of *labA* was CheA/CheY dependent, indicating that these two adhesins use different signaling pathways to sense lactate.

Only a small population of *H. pylori* bacteria directly bind to gastric epithelial cells, whereas most reside in the mucus layer overlying the cells and are attached to highly glycosylated mucins^39^. The expression of the major mucin in the stomach, MUC5AC, has been shown to increase during *H. pylori* infection, and the bacterium binds to MUC5AC through its adhesin BabA and lacdiNAc-binding adhesin (LabA), which specifically bind to lacdiNAc structures present on MUC5AC^12,40^. Here, we demonstrate that the expression of *labA* in *H. pylori* was downregulated during incubation of the bacteria in lactate. Both stereoisomers of lactate were able to reduce the expression of this adhesin.

*H. pylori* expresses several surface adhesins that lead to persistent colonization of the human stomach. The best characterized *H. pylori* adhesin, SabA, contributes to bacterial pathogenesis by mediating attachment to sialylated glycans on host cells during chronic inflammation^10,41^. Upon *H. pylori* infection, the expression of sialylated glycans increases in infected tissue, which makes SabA the adhesin of choice in the bacterium^5^. We showed that both l- and d-lactate inhibit *sabA* mRNA expression in *H. pylori*. The bacterium uses different mechanisms to regulate the expression of its adhesin proteins*.* For instance, through phase variation (slipped-strand mispairing), a dinucleotide cytosine-thymine repeat in the coding region of *sabA* can result in altered tract length and switch the expression of *sabA* “on” and “off”^22,42^. SabA is also regulated by the two-component signal transduction system ArsRS, which responds to different environmental stimuli in the stomach. Acidic pH is one of the environmental cues that is recognized by the ArsRS system^21^. However, the pH was not affected in our experiment when lactate was present (Fig. S3). Interestingly, incubation of the *ars*S mutant with lactate did not reduce the expression of *sabA* compared to that of its isogenic wild-type strain, indicating involvement of the ArsRS two-component system in the regulation of *sabA* gene expression in the presence of lactate.

The lacdiNAc-binding adhesin LabA binds specifically to lacdiNAc present on the major mucin MUC5AC of the gastric mucosa. In contrast to the results for *sabA*, the lactate-mediated reduction in *labA* expression was not mediated by the ArsRS system. Instead, experiments comparing the wild-type strain with the *cheY* mutant showed involvement of the CheA/CheY system in the regulation of *labA* in the presence of lactate. Lactate is sensed by the *H. pylori* chemoreceptor TlpC, which in turn feeds into the chemotaxis signal transduction pathway governed by proteins involved in the CheA/CheY system^43^. However, the attractant response to lactate via the TlpC chemosensor has been shown to be strongest at a low concentration of 0.1 mM, while at higher concentrations of 1 and 10 mM, the responses are detectable and nondetectable, respectively^30^. It is possible that at concentrations of 0.5 mM and above, lactate is no longer an attractant to the TlpC receptor. Repellent and attractant-free receptors stimulate autophosphorylation of CheA, which results in phosphorylation of the response regulator CheY. CheY-P further interacts with proteins of the flagellar motor complex, which influences the swimming direction of the bacteria^24^. It has already been shown in other bacteria, such as *S. enterica* and *V. alginolyticus,* that chemotaxis-related genes control adhesion^44,45^. Additionally, in the case of *V. cholerae,* it has been speculated that the CheA/CheY system can indirectly mediate adhesion by controlling the secretion of intercellular polysaccharide adhesins^46^. This is in line with our results showing that the ability of *H. pylori* to downregulate *labA* in the presence of lactate is lost in the *cheY* mutant. In the future, it would be interesting to investigate the exact mechanism by which the CheA/CheY system regulates *labA*.

The expression of SabA is induced during inflammatory conditions, and adhesin has been shown to promote the attachment and activation of neutrophils, which are immune cells known to be key players in *H. pylori*-induced inflammation^47,48^. Neutrophils and other immune cells, such as macrophages, can have a damaging effect during chronic inflammation via the production of reactive oxygen and nitrogen species and proinflammatory cytokines. l-Lactate has been shown to reduce the levels of TNF and IL-6 in LPS-stimulated monocytes^49^, and our results in *H. pylori*-stimulated macrophages are in line with these reports. The blockage of certain adhesin genes and the inhibition of proinflammatory cytokines by lactate may represent a new mechanism by which the host inflammatory response is modulated during *H. pylori* infection.

In summary, our data demonstrate the anti-inflammatory effects of lactate on cytokine production in *H. pylori*-stimulated macrophages and the ability of lactate to suppress *sabA* and *labA* expression in the bacterium. SabA is one of the main virulence factors of *H. pylori* and promotes bacterial adhesion to mucins, host gastric epithelial cells and immune cells during inflammation. In the future, it would be intriguing to determine whether other *H. pylori* virulence genes are affected by the presence of lactate and to further characterize the lactate-mediated regulation of SabA and LabA.

## Materials and methods

### Bacterial strains and growth conditions

The *Helicobacter pylori* strain 67:21 has previously been described^50^. The *H. pylori* mutants 67:21Δ*arsS,* 67:21Δ*cheY* and 67:21Δ*tlpC* were designed and constructed in this study. All *H. pylori* were grown on Columbia blood agar plates (Thermo Fisher) supplemented with 8% defibrinated horse blood and 8% inactivated horse serum (Håtunalab) for 3 days at 37 °C under microaerophilic conditions, i.e., in an incubator with 5% O_2_, 10% CO_2_ and 85% N_2_. Before each experiment, bacteria from the agar plates were suspended in Brucella broth (Acumedia) containing 8% heat-inactivated fetal bovine serum (FBS) (Sigma Aldrich) and cultured for 24 h under shaking and microaerophilic conditions. At the time of experiment the medium was RPMI 1640 without glucose (Thermo Fisher). *Escherichia coli* strain DH5α (Invitrogen) was used in the construction of 67:21Δ*arsS,* 67:21Δ*cheY* and 67:21Δ*tlpC* and was cultured in LB medium at 37 °C. When the bacteria were required to be cultured on selective media, chloramphenicol was supplemented at the following concentrations: 5 μg/ml added to CBA plates for *H. pylori* and 10 μg/ml added to LB plates for *E. coli.* *H. pylori* strain 26695 (ATCC 700392) and J99 (ATCC 700824) have previously been described and was used to verify results as compared to strain 67:21.

### Cell lines and culture conditions

The monocytic cell line THP-1 (ATCC TIB-202) was also purchased from ATCC. The cell lines were cultured in RPMI 1640 (Thermo Fisher) supplemented with 10% FBS. The cells were maintained at 37 °C and 5% CO_2_ in a humidified environment. THP-1 cells were induced to differentiate into macrophages using 0.1 μM phorbol 12-myristate 13-acetate (PMA) (Sigma Aldrich) for 3 days.

### Quantitative PCR assays

Liquid cultures of *H. pylori* grown overnight in Brucella broth, as specified above, were centrifuged at 4500×*g* for 10 min, and the resulting bacterial pellets were suspended in RPMI 1640 medium without glucose to an optical density of 0.3. This bacterial suspension was incubated in the absence or presence of different concentrations of l- or d-lactate (0.1 mM, 0.3 mM, 0.5 mM, 5 mM or 50 mM) for 2 h, based on changes in gene expression in this strain of *Heliocbacter pylori* 67:21 at a 2 h time point that have been previously studied^51^. The bacteria were pelleted by centrifugation at 4500×*g* for 10 min and treated with RNAprotect Bacteria Reagent (Qiagen) for 5 min at room temperature. The suspension was centrifuged at 5000×*g* for 10 min, and the resulting bacterial pellets were resuspended in lysis buffer (30 mM Tris–HCl, 1 mM EDTA, 15 mg/ml lysozyme (Sigma Aldrich) and proteinase K (Qiagen) and incubated for 1 h at room temperature, with 10 s of vortexing with 2 min rest cycles for the first half hour. RNA was isolated using an RNeasy kit (Qiagen) according to the manufacturer’s instructions. The RNA yield and purity were analyzed with a NanoDrop 8000 spectrophotometer, and the RNA was reverse transcribed into cDNA using SuperScript VILO Mastermix (Thermo Fisher). One µg of RNA was used for reverse transcription into cDNA and 1 µg total cDNA was used for the quantitative PCR (qPCR) of each sample. qPCR was performed using a LightCycler 480^10^ system and SYBR Green 1 mastermix^10^. The primers were specific for each gene of interest and are listed in Table 1 and have been tested for PCR efficiency. The qPCR program was as follows: initial denaturation of 95 °C for 10 s; annealing at 55 °C for 20 s; and extension for 72 °C for 20 s. The melt curve analysis was as follows; 95 °C for 5 s, 65 °C for 1 min, and then increased to 95 °C for 0.08 °C/s. qPCR negative controls containing all components of the reaction mixture except the cDNA template were included to check for nucleic acid contamination and primer dimer formation. The expression was analyzed by the 2(−ΔΔCt) method and normalized against the housekeeping gene *gyrB,* and the expression levels are given relative to those in the control samples. As an additional control, we used another house-keeping gene, *glmM*, confirming the results (data not shown). Treatment of samples with DNase I (New England Biolabs) were done as control, and did not change the results. As control, we measured the total RNA concentrations after overnight incubation in Brucella broth and after 2 h in RPMI, and they were similar.

**Table 1:** Primers used in this study.

Gene	Primer sequence (5′–3′)
gyrase B	Forward: CGTGGATAACGCTGTAGATGAGAGC
	Reverse: GGGATTTTTTCCGTGGGGTG
sabA	Forward: TGAAACGCCACTGATGACGA
	Reverse: AACCGTGAGCAACGCTCTTA
babA	Forward: CGATACCCTGGCTCGTTGTT
	Reverse: GGTTTTGGAATGTCGTGGGC
horB	Forward: GTGGGATTCGCTTAGGCACT
	Reverse: TAAAAGGCATAGGGGCGGTG
alpA	Forward: CACGCACTTCCAGTTCCTCT
	Reverse: ACTACGCCAAATTCCACCGT
alpB	Forward: GCCGGTAACAGCTCTCAAGT
	Reverse: AAGCCGTAGTAGCGTAAGCC
cagL	Forward: CTCAAAGCAATGGCCGCTTT
	Reverse: AGACCAACCAACAAGTGCTCA
hopZ	Forward: ATCGCACCGTTGTTGGTTTG
	Reverse: GGGGCTTACAGGCCGTTTAT
oipA	Forward: TAACGATAAGCGAGCGTCCC
	Reverse: TTAGCGTCTAGCGTTCTGCC
labA	Forward: GCTCATGACTTGCCACAACC
	Reverse: CCGACCCCAACGCTATCAAT
arsS up	Forward: GGCATTAGTGCGGCTAACACACAAAAT
	Reverse: ACTGATTTAGTGTATGATGGAACCCCTTAACTCCTTATTAGAAT
arsS down	Forward: ATAATAAGCGGATGAATGGCAGAAAAACAAAAAGAGAGAACATG
	Reverse: TTAGTGGAATAACTCATGATGGGCGTGT
arsS Cmr	Forward: GGAGTTAAGGGGTTCCATCATACACTAAATCAGTAAGT
	Reverse: CTTTTTGTTTTTCTGCCATTCATCCGCTTATTATCACT
cheY up	Forward: CGTGCCTATTTGATAAGAAGAAGACAT
	Reverse: TAGTGTATGATGGAGCGCTTCTCCTTTTAAGATTGCAT
cheY down	Forward: GCGGATGAATGGCGTGTTAAAGCCAATGTATTATGAGTT
	Reverse: CTTCTAGCCAGTCTTTTGAAGCCAGAT
cheY Cmr	Forward: AAGGAGAAGCGCTCCATCATACACTAAATCAGTAAGTT
	Reverse: GGCTTTAACACGCCATTCATCCGCTTATTATCACT
tlpC up	ATGAAAGTCATGCAGCCCTTGCTT
tlpC down	GCGATAGTCAAATTATCCTGCACAT

### *Construction of tlpC, arsS *and *cheY* deletion mutants

The donor DNA *arsS* and *cheY* deletion mutant (Δ*arsS* and Δ*cheY*) constructs were generated by combining the DNA upstream of the target gene amplified from the genome of *H. pylori* 67:21 via an upstream primer pair for the target gene, the chloramphenicol resistance cassette amplified from the plasmid pACYC184 using the Cmr primer pair, and the DNA downstream of the target gene amplified by a downstream primer pair for the target gene via overlapping PCR using high-fidelity Phusion DNA polymerase (Thermo Fisher Scientific) (see Table 1 for primers), while amplification of each of the fragments individually was performed using GoTaq^®^ G2 Flexi DNA Polymerase (Promega Biotech). The fusion products were inserted into pJET1.2/blunt cloning vectors (Thermo Fisher Scientific), and the ligation mixtures were used to transform *E. coli* DH5α. For construction of the *tlpC* deletion mutant (Δ*tlpC*), a custom-made pUC57-Mini plasmid vector containing a chloramphenicol resistance cassette inserted within the functional *tlpC* gene sequence was ordered from GenScript and used to transform *E. coli* DH5α. The respective plasmids were then isolated, and the *H. pylori* 67:21 mutants were created via electroporation of wild-type *H. pylori* 67:21 with the respective plasmids and selection for chloramphenicol resistance as described previously^52^. The generation of the desired mutants with replacement of the target genes by the antibiotic resistance cassette was confirmed by PCR and as well as sequencing (Eurofins). “Generation of complemented mutants were unsuccessful. Lowest possible antibiotic concentrations were used for selection to avoid double mutants, and appropriate phenotypes were confirmed, *i.e.,* lack of motility for ∆*cheY* mutant and acid sensitivity for ∆*arsS*.”

### ELISA

Differentiated THP-1 cells in 24-well plates were infected with *H. pylori* at an MOI of 100 in the presence or absence of l-lactate or d-glucose (0.5 mM, 5 mM or 50 mM) for 2 and 6 h. The secreted levels of the cytokines TNF and IL-6 were quantified in the cell culture supernatants using ELISA kits (Biolegend) by following the manufacturer’s instructions. The absorbance was measured using a Spectramax i3x microplate reader at 450 nm (with a reference wavelength of 570 nm).

### Flow cytometry

Bacteria were stained with 3 µg/µl CFSE CellTrace™ Cell Proliferation Kit stain (Invitrogen) for 15 min under shaking conditions, followed by incubation with FBS under the same conditions to inactivate the unbound stain. The bacteria were then washed using PBS and used to infect differentiated THP-1 cells in 24-well plates at an MOI of 100 in the presence or absence of l-lactate (0.5 mM, 5 mM or 50 mM). After 6 h of incubation, the samples were washed twice with PBS and detached from the wells by incubation with trypsin for 5 min at 37 °C before fixation with 4% PFA. At the time of analysis, each sample was treated with 0.2% trypan blue for 1 min to quench the fluorescent signal from the extracellular bacteria before analyzing the samples via flow cytometry using an BD LSRFortessa flow cytometer (BD Biosciences). For each sample, 30,000 cells were counted. Data were acquired using the BD FACSDiva software v8.0.1 analyzed with FlowJo software v10.8.1.

### Statistical analysis

Statistical significance was determined using GraphPad Prism software (version 9). Analysis of variance (ANOVA) followed by a Bonferroni posttest was used to compare differences between groups. *P* values less than 0.05 were considered statistically significant. Error bars represent the standard deviation.

## Supplementary Information

Supplementary Figures.

## Data Availability

The datasets used and/or analyzed in the current study are available from the corresponding author on reasonable request.
