# Supplementary material for: Lactate causes downregulation of Helicobacter pylori adhesin genes sabA and labA while dampening the production of proinflammatory cytokines
Source: Sci Rep. 2022 Nov 21;12:20064. doi: 10.1038/s41598-022-24311-5 (PMC9681763; doi:10.1038/s41598-022-24311-5)
Supplement: Supplementary file 1 — Supplementary Figures. [file 41598_2022_24311_MOESM1_ESM.pdf]

Supplementary Material

Lactate downregulates *Helicobacter pylori* adhesin genes *sabA* and *labA* while dampening the production of pro-inflammatory cytokines

Tanvi Somiah, Hanna Gebremariam, Fanglei Zuo, Ksenija Smirnova and Ann-Beth Jonsson*

Corresponding author: Ann-Beth Jonsson, E-mail: *ann-beth.jonsson@su.se*

Figure S1

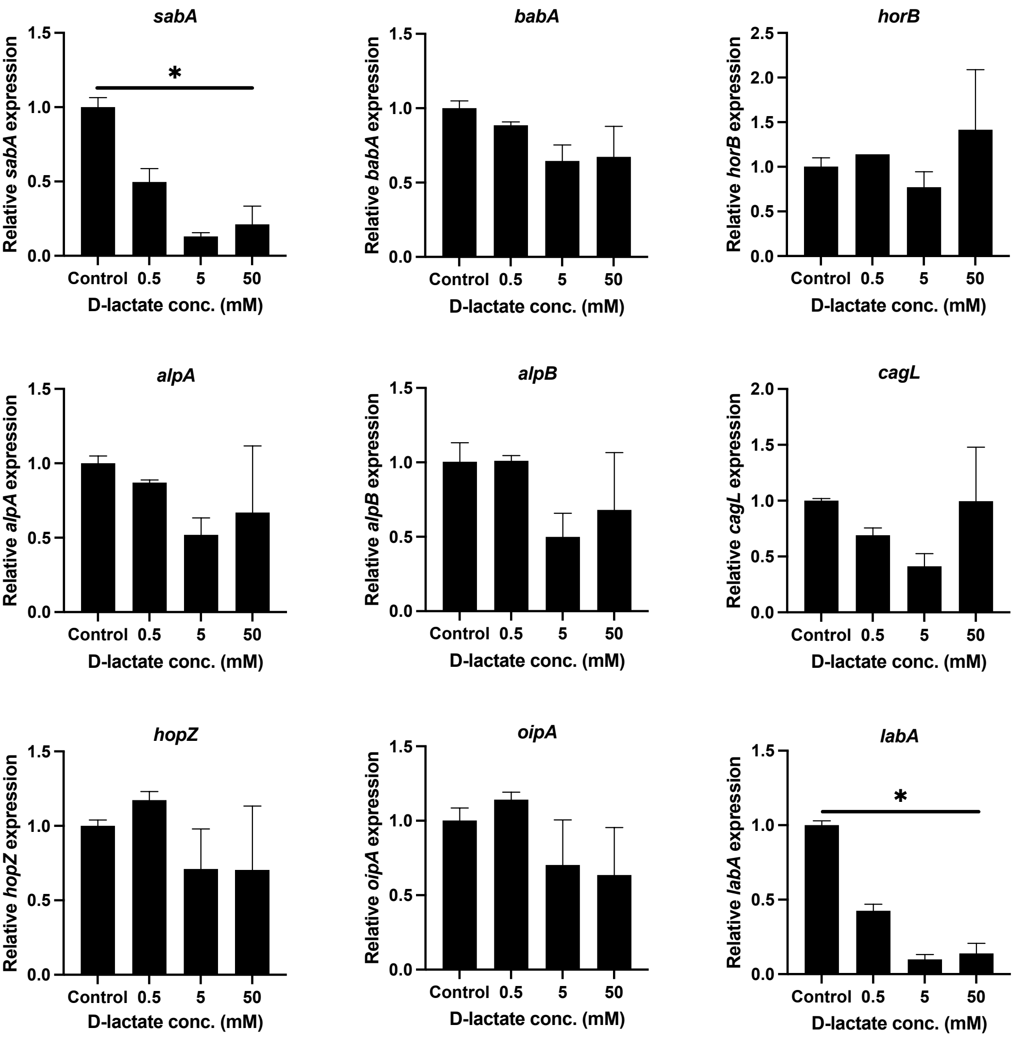

Figure S1. D-Lactate inhibits *sabA* and *labA* expression in *H. pylori*

mRNA expression levels of different *H. pylori* colonization-associated genes after incubation of the bacteria in RPMI 1640 cell culture medium without glucose (control) or supplemented with various concentrations of D-lactate for 2 h. Target gene expression was normalized to that of the housekeeping gene *gyrB*. Gene expression levels in the control were set to 1. Data represent the means and standard deviation of duplicate samples and are representative of three independent experiments. *P < 0.05; ns, nonsignificant, using ANOVA followed by a Bonferroni posttest.

.

Figure S2

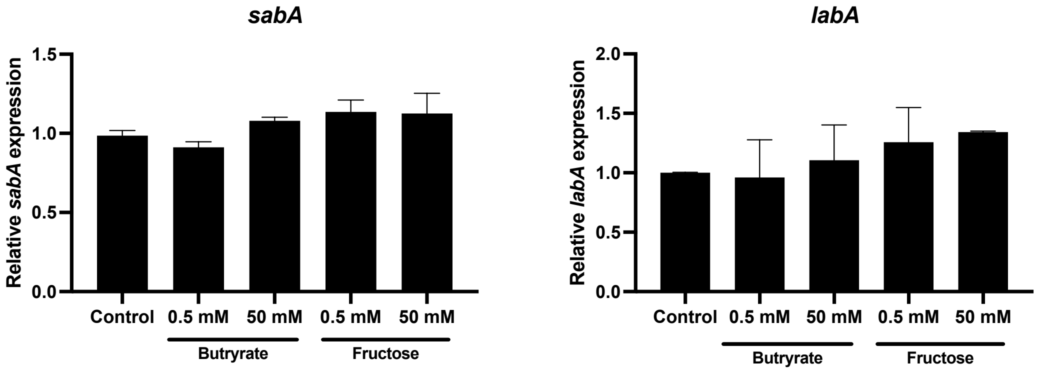

Figure S2. Fructose and butyrate do not cause the downregulation of *sabA* and *labA*, indicating that the response is lactate-specific

Gene expression of *H. pylori* adhesins *sabA* and *labA* after incubation of the bacteria in RPMI 1640 medium without glucose (control) or supplemented with various concentrations of butyrate and fructose. mRNA expression levels of various *H. pylori* adhesin genes were quantified using qPCR after 2 h. Target mRNA levels were normalized to those of the housekeeping gene *gyrB*. Gene expression levels in the control were set to 1. Data represent the means and standard deviation of duplicate samples and an average of two independent experiments, using ANOVA followed by a Bonferroni posttest.

.

Figure S3

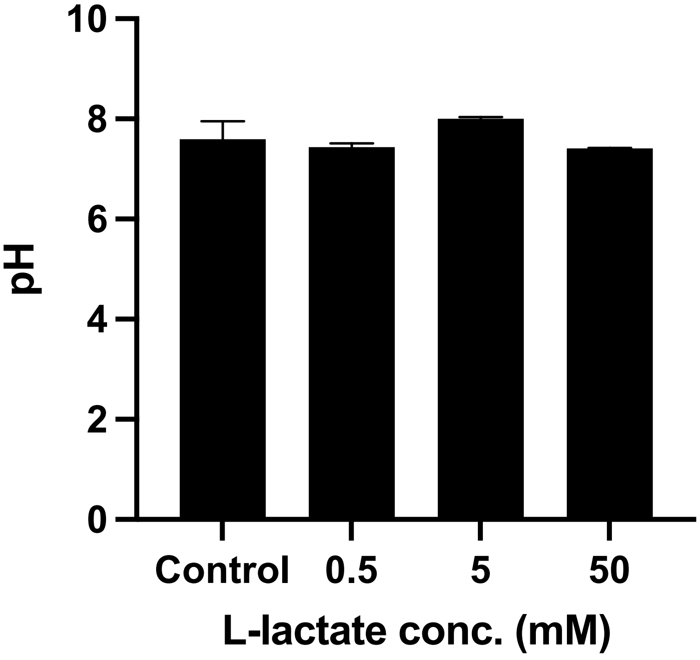

Figure S3. Effect of lactate on the pH of the medium

The pH of *H. pylori* samples after incubation in RPMI 1640 cell culture medium without glucose (control) or supplemented with various concentrations of L-lactate for 2 h. Data represent the means and standard deviation of duplicate samples and are representative of two independent experiments, using ANOVA followed by a Bonferroni posttest.

.

Figure S4

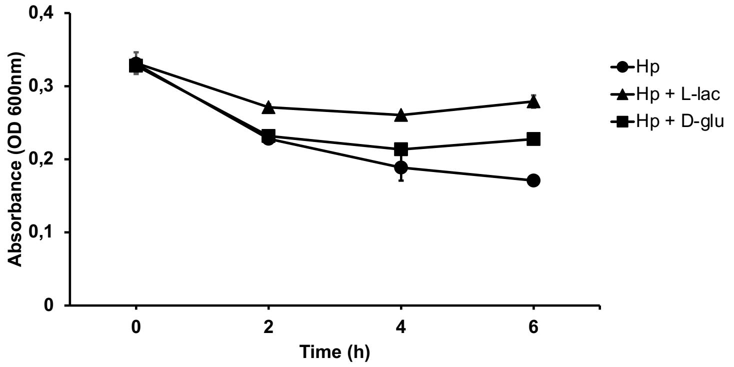

Figure S4. ***H. pylori*** **grows at a similar level in media containing L- lactate, D-glucose or neither supplement**

H. pylori was grown in RPMI 1640 medium without glucose, supplemented with 5 mM L-lactate, 5 mM D-glucose or neither supplement, and cultured for up to 6 h. O.D 600 readings were taken at 2, 4 and 6 h. Data represent the means and standard deviation of duplicate samples and are representative of three independent experiments, using ANOVA followed by a Bonferroni posttest.
